# Complexity of the eukaryotic dolichol-linked oligosaccharide scramblase suggested by activity correlation profiling mass spectrometry

**DOI:** 10.1038/s41598-020-80956-0

**Published:** 2021-01-14

**Authors:** Alice Verchère, Andrew Cowton, Aurelio Jenni, Monika Rauch, Robert Häner, Johannes Graumann, Peter Bütikofer, Anant K. Menon

**Affiliations:** 1 Department of Biochemistry, Weill Cornell Medical College, 1300 York Ave, New York, NY 10065 USA; 2 Institute of Biochemistry and Molecular Medicine, University of Bern, Bühlstr. 28, 3012 Bern, Switzerland; 3 Graduate School for Cellular and Biomedical Sciences, University of Bern, Mittelstr. 43, 3012 Bern, Switzerland; 4 Department of Chemistry and Biochemistry, University of Bern, Freiestr. 3, 3012 Bern, Switzerland; 5 Max Planck Institute for Heart and Lung Research, W.G. Kerckhoff Institute, Ludwigstr. 43, 61231 Bad Nauheim, Germany; 6 German Centre for Cardiovascular Research (DZHK), Rhine-Main site, Max Planck Institute for Heart and Lung Research, Bad Nauheim, Germany

**Keywords:** Biochemistry, Biological techniques, Cell biology, Microbiology

## Abstract

The oligosaccharide required for asparagine *(N)*-linked glycosylation of proteins in the endoplasmic reticulum (ER) is donated by the glycolipid Glc_3_Man_9_GlcNAc_2_-PP-dolichol. Remarkably, whereas glycosylation occurs in the ER lumen, the initial steps of Glc_3_Man_9_GlcNAc_2_-PP-dolichol synthesis generate the lipid intermediate Man_5_GlcNAc_2_-PP-dolichol (M5-DLO) on the cytoplasmic side of the ER. Glycolipid assembly is completed only after M5-DLO is translocated to the luminal side. The membrane protein (M5-DLO scramblase) that mediates M5-DLO translocation across the ER membrane has not been identified, despite its importance for *N*-glycosylation. Building on our ability to recapitulate scramblase activity in proteoliposomes reconstituted with a crude mixture of ER membrane proteins, we developed a mass spectrometry-based 'activity correlation profiling' approach to identify scramblase candidates in the yeast *Saccharomyces cerevisiae*. Data curation prioritized six polytopic ER membrane proteins as scramblase candidates, but reconstitution-based assays and gene disruption in the protist *Trypanosoma brucei* revealed, unexpectedly, that none of these proteins is necessary for M5-DLO scramblase activity. Our results instead strongly suggest that M5-DLO scramblase activity is due to a protein, or protein complex, whose activity is regulated at the level of quaternary structure.

## Introduction

**Figure 1:**
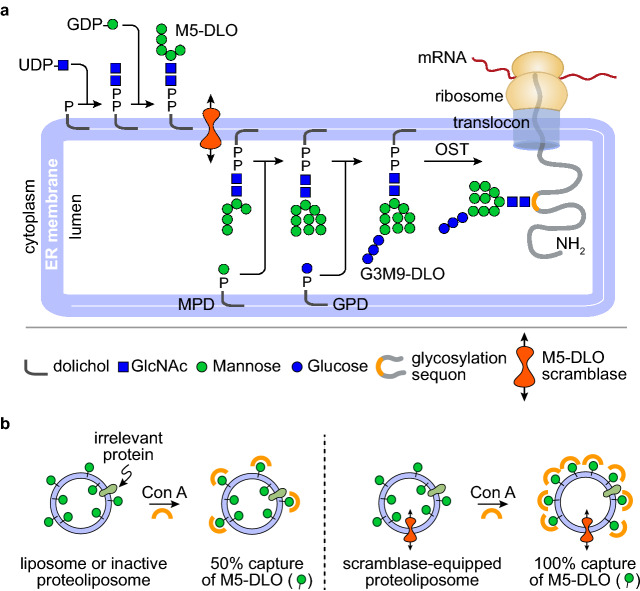
Transbilayer movement of Man_5_GlcNAc_2_-PP-dolichol (M5-DLO). (** a**) Synthesis of Glc_3_Man_9_GlcNAc_2_-PP-dolichol (G3M9-DLO), the oligosaccharide donor for protein *N*-glycosylation in yeast and humans, occurs in two stages. The first stage, comprising seven glycosyltransfer reactions, occurs on the cytoplasmic side of the ER to generate M5-DLO from dolichyl phosphate, UDP-GlcNAc and GDP-mannose. The remaining seven glycosyltransfer reactions occur in the ER lumen, adding four mannose residues (from mannose phosphate dolichol (MPD)) and three glucose residues (from glucose phosphate dolichol (GPD)) to M5-DLO to generate G3M9-DLO. The switch from the cytoplasmic to the lumenal side of the ER requires transbilayer movement of M5-DLO, which is facilitated by M5-DLO scramblase. The right side of the figure shows oligosaccharyl-transferase (OST)-mediated transfer of the Glc_3_Man_9_GlcNAc_2_ oligosaccharide from G3M9-DLO to an asparagine residue within a glycosylation sequon in a nascent protein as it emerges from the protein translocon into the ER lumen. See text for details. (**b**) Recapitulation of M5-DLO scrambling in a reconstituted system. The assay exploits the organic-solvent-resistant interaction between M5-DLO and the lectin Concanavalin A (Con A)—thus whereas M5-DLO is soluble in organic solvent, M5-DLO/ConA complexes are precipitated. Proteoliposomes are reconstituted from egg phosphatidylcholine, [^3^H]M5-DLO and ER membrane proteins. Protein-free liposomes are prepared in parallel. Incubation of protein-free liposomes with Con A results in the capture/precipitation of ~ 50% of [^3^H]M5-DLO (left), corresponding to the pool in the outer leaflet of the vesicles, whereas similar incubation of proteoliposomes containing M5-DLO scramblase results in the capture of ~ 100% of [^3^H]M5-DLO (right) as M5-DLO molecules in the inner leaflet are translocated to the outer leaflet where they are captured by Con A. Proteoliposomes reconstituted with proteins other than the M5-DLO scramblase, i.e. irrelevant proteins, are expected to behave similarly to protein-free liposomes (left).

Asparagine *(N)*-linked protein glycosylation is found in all domains of life^1–5^. In eukaryotes, it takes place in the lumen of the endoplasmic reticulum (ER) via oligosaccharyltransferase (OST)-mediated *en bloc* transfer of a pre-synthesized oligosaccharide (Glucose_3_Mannose_9_*N*-acetylglucosamine_2_ (Glc_3_Man_9_GlcNAc_2_) in yeast and humans) to specific asparagine residues in newly translocated proteins^6,7^. The Glc_3_Man_9_GlcNAc_2_ oligosaccharide is built by sequentially adding sugars to dolichyl phosphate, an isoprenoid glycosyl carrier lipid with a very long hydrocarbon chain (Fig. 1a). Sugar addition to dolichyl phosphate occurs in two stages that take place on different sides of the ER membrane. The first stage comprises seven reactions in which the sugar nucleotide donors UDP-GlcNAc and GDP-Man are used to produce the key intermediate Man_5_GlcNAc_2_-PP-dolichol (termed Man_5_GlcNAc_2_ dolichol-linked oligosaccharide, or M5-DLO) on the cytoplasmic face of the ER (Fig. 1a). M5-DLO is then translocated across the ER membrane to the luminal side where seven glycosyltransfer reactions convert it to Glc_3_Man_9_GlcNAc_2_-PP-dolichol (G3M9-DLO). The mannose and glucose residues needed for these latter reactions are donated by the glycolipids mannose phosphate dolichol (MPD) and glucose phosphate dolichol (GPD), both of which are synthesized on the cytoplasmic face of the ER and flipped to the luminal leaflet^8–14^.

The assembly of G3M9-DLO thus requires the rapid translocation of three polar glycolipids—M5-DLO, MPD and GPD—from the cytoplasmic to the luminal leaflet of the ER^3,15^. Biochemical assays provide compelling evidence that these translocation events do not require metabolic energy, i.e. they are ATP-independent, and that they are mediated by ER membrane proteins (termed scramblases) with exquisite substrate specificity^8–10,16,17^. The ER membrane protein Rft1 was proposed as the M5-DLO scramblase almost two decades ago^18,19^, but subsequent work showed that whereas Rft1 is clearly important for *N*-glycosylation, it appears to have no direct role in translocating M5-DLO across the ER membrane^16,20–23^. Likewise, the mammalian protein Lec35/MPDU1 appeared to be involved in MPD scrambling, but likely in the role of a 'dolichol chaperone' that may assist in an as yet undefined way in the scrambling process^11,12^. Thus, the molecular identity of the scramblase proteins required to transfer M5-DLO, MPD and GPD across the ER membrane remains unknown^15,24^. Here we focus on the M5-DLO scramblase.

Unlike the MPD and GPD scramblases, M5-DLO scramblase is critically important as *N*-glycosylation cannot occur without it. We developed a method to assay M5-DLO scramblase activity in large unilamellar vesicles reconstituted with a mixture of ER membrane proteins derived from yeast or rat liver. We used radiolabeled M5-DLO as the reporter lipid, and the α-mannosyl-binding lectin Concanavalin A as a topological probe^16,17,20,25,26^. The assay is described in detail in Fig. 1b. Using this assay, we showed that the M5-DLO scramblase activity is ATP-independent, and highly structure specific ^15^. Thus, higher order structures such as M7-DLO and M9-DLO are not scrambled efficiently ^16^, nor is a structural isomer of M5-DLO in which the mannose residues correspond to the arrangement seen in 'processed *N*-glycans'^17^. We could also resolve M5-DLO and MPD scramblase activities^10,16^. For example, by fractionating detergent-solubilized ER membrane proteins on Con A-Sepharose resin and reconstituting the flow-through and eluted fractions in liposomes to assay scramblase activity, we demonstrated that MPD scramblase is not a glycoprotein whereas M5-DLO scramblase is either a glycoprotein or a component of a heteromeric complex that contains a glycoprotein. Despite these considerable advances, a straightforward purification of the scramblases proved elusive. To circumvent the problem of protein purification we therefore conceptualized a quantitative proteomics approach. Here we describe this approach and its application to the problem of identifying the M5-DLO scramblase.

## Results

### Identifying M5-DLO scramblase candidates by activity correlation profiling

**Figure 2:**
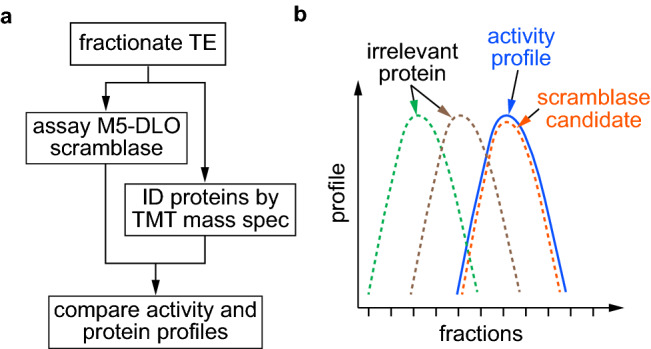
Principle of the activity correlation profiling approach. (** a**) A Triton X-100 extract (TE) of salt-washed yeast membranes, i.e. an extract that is enriched in ER membrane proteins, is fractionated by velocity gradient sedimentation. Half of each fraction is reconstituted into large unilamellar vesicles and assayed for M5-DLO scramblase activity as outlined in Fig. 1b to generate a profile of scramblase activity across the gradient. The remaining half of each fraction is subjected to mass spectrometric analysis using the tandem mass tag (TMT) system. Thus, each fraction is digested with trypsin, and the resulting peptides are labeled with TMT multiplex reagents (a unique TMT mass tag is used for each fraction). The digests are pooled and analyzed by tandem mass spectrometry to quantify the relative amount of identified proteins across fractions, thereby generating protein profiles. The protein profiles are quantitatively compared with the activity profile by obtaining a Pearson correlation score. (**b**) Schematic illustration of the readout from the activity correlation profiling experiment. The activity profile (solid blue line) can be compared to the profile of several different proteins (dashed lines) identified by tandem mass spectrometry. Only a protein whose profile is highly correlated with the activity profile (orange dashed line) is considered as a scramblase candidate. Other proteins that fractionate differently (brown and green dashed lines) are considered irrelevant to scramblase activity.

Hypothesizing that M5-DLO scramblase activity is due to a single protein or unique protein complex, present as a homogeneous entity in the ER, we developed a mass spectrometry-based activity correlation profiling approach^27,28^ to identify scramblase candidates from a crude mixture of detergent-solubilized ER membrane proteins (Fig. 2). The underlying concept involves resolving the proteins in the crude mixture into a number of fractions using any separation technique of choice, measuring the scramblase activity of each fraction to generate an activity profile and, in parallel, using mass spectrometry to determine the relative abundance of individual proteins across the fractions (Fig. 2a). Proteins whose profiles best match the activity profile (Fig. 2b) are filtered through a stringent data curation process to identify scramblase candidates.

Total membranes from a yeast cell homogenate were washed with high salt to remove peripheral proteins, and then treated with ice-cold Triton X-100 to extract selectively ER membrane proteins as described previously^16^. The resulting 'Triton Extract' (TE) was fractionated by velocity gradient sedimentation using a continuous glycerol gradient. Sedimentation standards were analyzed in a parallel gradient. Gradient fractions were collected manually from the top and, after measurement of refractive index, the fractions were passed over a desalting spin column to remove glycerol. A portion of each fraction from the middle section of the gradient (fractions 5–11, shown in preliminary tests to contain the majority of M5-DLO scramblase activity) was reconstituted into large unilamellar vesicles and assayed for scramblase activity using [^3^H]M5-DLO (Fig. S1), whereas the remainder was taken for mass spectrometry using 6-plex tandem mass tag (TMT) reagents^29^.

The M5-DLO scramblase activity assay has been described in detail previously^16,17,20^. The assay (Fig. 1b) exploits the organic-solvent resistant interaction between M5-DLO and the lectin Concanavalin A (Con A) to capture M5-DLO molecules located on the external surface of large unilamellar vesicles, and thereby separate them from those located in the inner leaflet. In the absence of a reconstituted functional scramblase, M5-DLO molecules located in the inner leaflet of the vesicles are inaccessible to Con A, whereas if the vesicles contain a scramblase then these molecules are translocated from the inner leaflet to the external leaflet and captured by the lectin. A key point is that the rate of scrambling is greater than the rate at which M5-DLO is captured by Con A^16,17^, even if the assay is conducted on ice, which would be expected to reduce the scrambling rate. Thus, the assay reports end-point data, ranging theoretically from 50 to 100% M5-DLO captured depending on the proportion of vesicles in the sample that is reconstituted with a functional M5-DLO scramblase. The reconstitution of M5-DLO scramblase molecules into an ensemble of lipid vesicles is governed by Poisson statistics (for example, see references^30–32^). To know the amount of M5-DLO scramblase in a mixture of proteins, e.g. in a fraction from the velocity gradient, we would therefore have to carry out reconstitutions with a range of protein concentrations and use a Poisson model to calculate scramblase abundance. As this is an impractical undertaking when assaying fractions from the velocity gradient, we decided instead to estimate scramblase abundance by reconstituting just enough protein so that the fraction with the highest amount of M5-DLO scramblase would report approximately 70% M5-DLO capture. We determined empirically that this could be accomplished by reconstituting half of each fraction.

**Figure 3:**
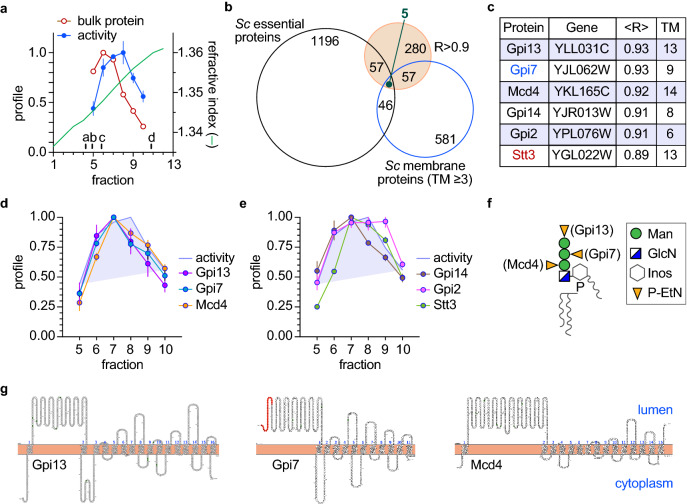
Identification of M5-DLO scramblase candidates by activity correlation profiling. (**a**) Yeast TE was fractionated by velocity sedimentation on a 5–25% glycerol gradient and fractions were collected from the top. Sedimentation standards (indicated as a-d: 2.8, 3.6, 4.2 and 8.9S) were resolved in parallel. The profiles of bulk protein (open circles) and M5-DLO scramblase activity (solid circles, duplicate measurements) are indicated, along with the refractive index (green line) of the fractions. (**b**) Venn diagram describing the selection of M5-DLO scramblase candidates. Mass spectrometry identified 280 proteins with a correlation coefficient R > 0.9 (filled circle). Intersections between this group and the groups of all essential proteins in yeast (*Sc* essential proteins), and yeast membrane proteins with three or more predicted transmembrane spans (*Sc* membrane proteins (TM ≥ 3, assessed by the TMHMM membrane protein topology prediction program)) yielded 5 proteins. One of these (Neo1) was discarded because it is not an ER resident. (**c**) List of M5-DLO scramblase candidates. The four ER membrane proteins obtained at the intersection of the Venn diagram are listed. Gpi7 and Stt3 were included for reasons discussed in the text. (**d**) Profiles of Gpi13, Gpi7 and Mcd4 vs the activity profile (shaded and outlined in blue). See also Fig. S4. (**e**) Profiles of Gpi14, Gpi2 and Stt3 vs the activity profile (shaded and outlined in blue). (**f**) Role of Gpi13, Gpi7 and Mcd4 in GPI P-EtN transfer reactions. The structure of the mature GPI anchor precursor in yeast and mammals is shown. Mcd4, Gpi7 and Gpi13 add the first, second and third P-EtN residues, respectively. *T. brucei* possesses Gpi13 but lacks homologs of Mcd4 and Gpi7. (**g**) Membrane topology of Gpi13, Gpi7 and Mcd4 assessed using Protter^67^ and placed in the context of the ER membrane (horizontal bar). A predicted signal sequence at the N-terminus of Gpi7 is indicated in red. The number of predicted transmembrane spans differs slightly from the number predicted by the TMHMM server that was used to calculate the Venn diagram group '*Sc* membrane proteins (TM ≥ 3)'.

The quality of separation achieved on the velocity gradient is shown in Fig. 3a. The activity profile has a peak in fractions 7–8, corresponding to a nominal sedimentation coefficient of 6-7S, whereas the bulk of the protein peaks earlier in the gradient, in fraction 6. Approximately 3000 proteins were identified in the mass spectrometric analysis (Tables S1 and S2). To learn how the various proteins were resolved on the gradient we determined the fraction in which each protein had its maximum abundance. This allowed us to obtain the average molecular weight of proteins that had their maximum abundance in each fraction. The data show that this average molecular weight increases from ~ 40 kDa (fraction 5) to ~ 80 kDa (fraction 8), remaining approximately constant at ~ 80 kDa over fractions 8–10 (Fig. S2). This result is consistent with the general expectation that protein monomers and small complexes will predominate in the initial fractions, and higher order complexes will be found in fractions 8–10. In line with this conclusion, the multi-subunit OST complex and the glycosyl-phosphatidylinositol (GPI) transamidase complex sediment relatively rapidly in the gradient, with the majority of their constituent subunits being maximally detected in fraction 7 (Fig. S3).

We next quantitatively compared the protein abundance profiles (obtained from each of two technical replicates (Tables S1 and S2)) with the scramblase activity profile, identifying 280 proteins whose profile matched that of the activity profile with a correlation score R ≥ 0.9 (Fig. S4). We subjected this list to several data curation steps. Hypothesizing that the *N*-glycosylation scramblase is an essential, ER-localized membrane protein, we discarded proteins that did not meet these criteria. Thus, 62 of the 280 proteins are known to be essential in yeast, and 5 correspond to ER-localized polytopic membrane proteins with 3 or more transmembrane spans as predicted by the TMHMM web server (Fig. 3b). These latter proteins are listed in Fig. 3c, and their profiles in comparison to the activity profile are shown in Figs. 3d, e and S4. We included the highly correlated but non-essential protein Gpi7 because it belongs to the same GPI phospho-ethanolamine-transferase (P-EtN) family as Mcd4 and Gpi13^33,34^ (Figs. 3f, S4) that were identified in our analysis; all three are polytopic membrane proteins with a large lumenal loop (Fig. 3g). Remarkably, the five candidates identified in our analyses are enzymes of the ER-localized GPI biosynthetic pathway^33^, and three of these (Gpi13, Gpi14, Gpi2) are ubiquitously found in eukaryotes. We noticed that the profile of Stt3, the catalytic subunit of OST (Fig. 1a), matched closely with the activity profile (correlation score of 0.89, close to our cut-off value of 0.9). Because of its importance to *N*-glycosylation we included Stt3 in our list of candidates.

### Biochemical test of M5-DLO scramblase candidates

To determine whether the six candidates (Gpi13, Gpi7, Mcd4, Gpi14, Gpi2 and Stt3) play a role in M5-DLO scrambling, we determined whether the M5-DLO scramblase activity present in TE could be eliminated by quantitatively and specifically immunodepleting the candidate from the TE prior to reconstitution and assay. We previously used a similar approach to show that opsin accounts for all the phospholipid scramblase activity in detergent-solubilized retinal disc membranes^35^, and that Rft1 does not contribute to M5-DLO scramblase activity in TE^20^.

**Figure 4:**
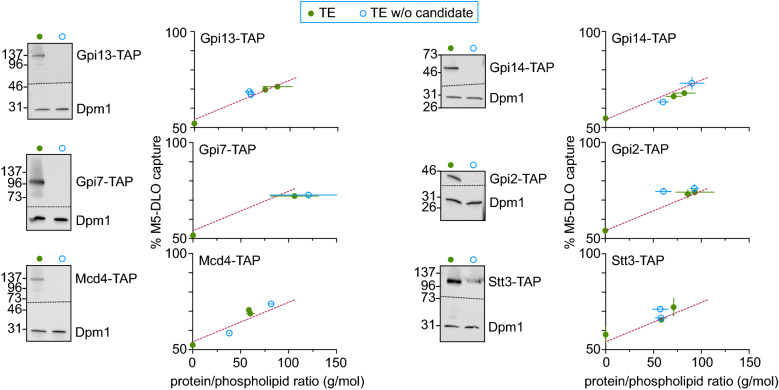
Evaluating M5-DLO scramblase candidates by immunodepletion. TE was prepared from yeast strains expressing a C-terminally TAP-tagged variant of the scramblase candidate in place of the endogenous protein. Tagging was done at the genomic locus. Six unique TE preparations were thus generated, corresponding to cells expressing Gpi13-TAP, Gpi7-TAP, Mcd4-TAP, Gpi14-TAP, Gpi2-TAP or Stt3-TAP. In every case the extract was mock-treated (green filled circles) or incubated with IgG-agarose beads to remove the TAP-tagged protein (blue open circles). Removal of the TAP-tagged protein from the extract was confirmed by SDS-PAGE/immunoblotting using anti-TAP antibodies, and specificity of immunodepletion and equivalence of sample loading was confirmed by immunoblotting to detect the ER resident membrane protein Dpm1. Mock-treated and depleted extracts were reconstituted and assayed for M5-DLO scramblase activity. Reconstitutions were performed at different ratios of protein to phospholipid, in the approximate range 25–125 g/mol. The figure shows immunoblots paired with the corresponding activity assays for each candidate. Error bars are obtained from duplicate measurements of activity, as well as duplicate measurements of the protein and phospholipid content of the reconstituted vesicles. The red dashed line, based on all data from the mock-treated samples, is intended to guide the eye. See Fig. S5 for uncropped versions of the immunoblots.

We obtained haploid yeast strains in which the M5-DLO scramblase candidate of interest is functionally expressed from its chromosomal locus with a C-terminal Tandem Affinity Purification (TAP) tag^36^. The TAP-tagged protein is thus the only version of that protein being expressed in the cell. TE was prepared from strains expressing individual TAP-tagged M5-DLO scramblase candidates and either mock-treated or treated with IgG resin to eliminate the TAP-tagged protein. Western blotting indicated that the TAP-tagged candidates were quantitatively removed by IgG-resin treatment, whereas an irrelevant ER membrane protein, the mannosyltransferase Dpm1, was still present in the TE (Figs. 4 and S5). We reconstituted equivalent amounts of mock-treated and IgG-treated TE into vesicles for M5-DLO scramblase activity assay. As our assay provides an end-point readout that reports on the proportion of vesicles that is equipped with a functional scramblase (see above), we expected to see a complete loss of activity for one of the candidates, thus pointing to its role in scrambling M5-DLO. Surprisingly, scramblase activity was not significantly affected by the removal of any of the candidates (Fig. 4). Thus, none of the candidates is necessary for the scramblase activity of TE as measured by our reconstitution-based assay system. However, as described below, we were nevertheless interested in further exploring the possible function of the GPI P-EtN transferases in M5-DLO scrambling.

### Further consideration of the GPI P-EtN transferase proteins as scramblase candidates

The three GPI P-EtN transferases have very similar membrane topology (Fig. 3g), carry out similar reactions (they each transfer P-EtN from phosphatidylethanolamine (PE)^37,38^ to mannose residues in the GPI core structure (Fig. 3f)), and are present at equivalent levels in the cell (Fig. S6). Previous reports suggest that these transferases are moonlighting proteins^39,40^, with additional functions in the cell distinct from their recognized roles in the GPI biosynthetic pathway. Thus, over-expression of any of the three proteins in yeast unexpectedly results in enhanced secretion of ATP, consistent with the possibility that each of these proteins is involved in the transport of ATP into the lumen of secretory compartments^41^. Furthermore, Mcd4 was shown to have a non-canonical role in aminophospholipid metabolism^42^ and ER protein quality control^43^, distinct from its role in GPI anchoring. Based on these indicators, we considered the possibility that the three GPI P-EtN transferases may each, redundantly, have M5-DLO scramblase activity. As the proteins are expressed at similar levels (Fig. S6), elimination of any one of them would result in a lowering of our scramblase assay read-out by one-third, whereas elimination of all three would result in total removal of scramblase activity from the TE. Although we did not see any significant difference in the assay readout when testing TE samples from which individual P-EtN transferases had been eliminated (Fig. 4), we considered the possibility that this could be a result of the potentially poor sensitivity of the assay to changes that are less than twofold. As described next, rather than continue with the reconstitution approach to explore a possible role of P-EtN transferases in M5-DLO scrambling, we took a genetic approach.

### In vivo test of Gpi13 as an M5-DLO scramblase

At the level of a single eukaryotic cell, *N*- glycosylation is essential for cell viability whereas GPI anchoring is generally not essential. Thus, mammalian cells in culture are non-viable when *N*-glycosylation is disrupted^6^, but their growth is unaffected by a deficiency in GPI anchoring^44^. The same holds true for the early diverging eukaryote *Trypanosoma brucei*^45–48^, the causative agent of African sleeping sickness. Interestingly, GPI anchors in *T. brucei* have only a single P-EtN, linked to the third mannose residue, that provides the means to attach the anchor to proteins^49–51^. Thus, *T. brucei* has an ortholog of Gpi13 (TbGPI13) but lacks orthologs of Mcd4 and Gpi7. To test whether Gpi13 has M5-DLO scramblase activity, we decided to generate TbGpi13 null mutants of insect stage (procyclic) *T. brucei*. We predicted that if TbGPI13 is solely involved in GPI anchoring, then *T. brucei* GPI13Δ cells would be viable. However, the *T. brucei* GPI13Δ cells would be inviable if TbGPI13 played an essential role in scrambling M5-DLO for protein *N*-glycosylation.

**Figure 5:**
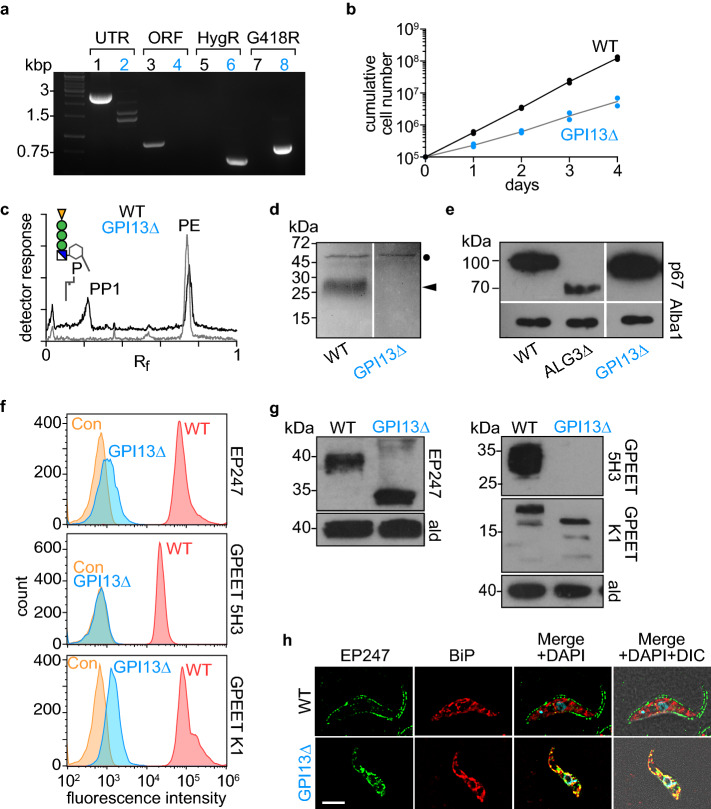
Evaluating Gpi13 as the M5-DLO scramblase in procyclic form *Trypanosoma brucei*. (** a**) PCR confirmation of *TbGPI13* gene replacement using primers specific to the *TbGPI13* UTR and ORF, and HygR/G418R gene replacement cassettes. Lanes 1, 3, 5 and 7 correspond to wild-type cells, whereas lanes 2, 4, 6 and 8 correspond to TbGPI13 knockout cells. (**b**) Growth of TbGPI13 knockout (GPI13Δ) and wild-type (WT) cells. Data points represent mean values from 2 independent experiments. (**c**) Thin layer chromatographic analysis of polar lipid extracts from [^3^H]-ethanolamine-labeled WT (top trace) and GPI13Δ (bottom trace) cells. The chromatograms were visualized using a radioactivity scanner. The WT trace is displaced upwards for clarity. PP1 and PE refer to the major GPI precursor in procyclic trypanosomes (structure shown in schematic) and PE, respectively. (**d**) SDS-PAGE/fluorography of protein extracts from [^3^H]-ethanolamine-labeled WT and GPI13Δ cells showing loss of GPI-anchored GPEET procyclin (indicated by arrow head) in GPI13Δ cells. The band at ~ 50 kDa (indicated by filled circle) represents eukaryotic elongation factor 1A, a protein that is modified by ethanolamine phosphoglycerol and therefore metabolically labeled with [^3^H]-ethanolamine. (**e**) Analysis of p67 *N*-glycosylation by anti-p67 immunoblot of whole cell protein samples from WT and GPI13Δ cells. A sample from a known *N*-glycosylation defective cell line (ALG3Δ) was used as a positive control. Alba1 was probed using anti-Alba1 antibody and used as a loading control. (**f**) Flow cytometry analysis of surface expression of GPI-anchored EP and GPEET procyclins in WT and GPI13Δ cells. The middle and bottom panels correspond to flow cytometry analysis of GPEET using 5H3 antibody (middle) and K1 antiserum (bottom). Control (Con) samples were generated by omitting the primary antibody. (**g**) Anti-EP and anti-GPEET (K1) procyclin immunoblots of whole cell samples from WT and GPI13Δ cells. Aldolase was probed using anti-aldolase antibody and used as a loading control. (**h**) WT and GPI13Δ cells were fixed and stained with antibodies against EP and BiP (as a marker for the endoplasmic reticulum) in combination with fluorophore-conjugated secondary antibodies and analyzed by fluorescence microscopy. DNA was stained with 4′,6-diamidino-2-phenylindole (DAPI) in the merged panels. DIC, differential interference contrast. Scale bar = 5 µm.

We sequentially disrupted the two alleles of TbGPI13 in procyclic-stage *T. brucei* using hygromycin and geneticin drug resistance cassettes (Fig. 5a), and recovered viable GPI13Δ cells that grew well, albeit approximately twofold more slowly than wild-type cells (Fig. 5b). Although our ability to generate viable GPI13Δ cells immediately indicates that Gpi13 does not play an essential role in *N*-glycosylation, we nevertheless characterized the cells to verify the expected disruption of GPI anchoring and to investigate the *N*-glycosylation status of specific glycoproteins. We performed metabolic radiolabeling with [^3^H]ethanolamine, and analyzed both polar lipids and proteins. Whereas both PE and the P-EtN-containing GPI anchor precursor PP1^51^ were labeled with [^3^H]ethanolamine in wild-type (WT) cells, PP1 was not labeled in GPI13Δ cells (Fig. 5c). Consistent with this observation, SDS-PAGE/fluorography of protein extracts showed radiolabeled GPI-anchored GPEET procyclin (arrowhead) in WT but not GPI13Δ cells (Fig. 5d). Of note, radiolabeling of eukaryotic elongation factor 1A, a protein that is modified by ethanolamine phosphoglycerol^52^, was similar in both WT and GPI13Δ cells (Fig. 5d).

We tested the intactness of the *N*-glycosylation pathway in GPI13Δ cells by assessing the glycosylation status of the lysosomal *N*-glycoprotein p67 which has 14 N-glycosylation sites. An anti-p67 immunoblot of whole cell protein samples from WT and GPI13Δ cells revealed no change in the molecular mass of p67, running at approximately 100 kDa, consistent with normal *N*-glycosylation^53^. In contrast, the mass of the glycoprotein was lower in a control *N*-glycosylation defective cell line (ALG3Δ) that cannot mannosylate M5-DLO *en route* to synthesis of the optimal DLO for the OST reaction^54^ (Fig. 5e).

Finally, we examined the status of non-GPI-anchored procyclins in the GPI13Δ cells. We found that the main consequence of the lack of GPI anchoring was that the proteins remain sequestered inside the cell, failing to exit the ER as evinced by flow cytometry and fluorescence microscopy (Fig. 5f–h).

Our results indicate that GPI anchoring is disrupted as expected in GPI13Δ cells, whereas *N*-glycosylation is unperturbed. The inability to synthesize GPI-anchored surface coat proteins may explain the somewhat slower growth rate of the cells^46,48^ (Fig. 5b). Taken together with the biochemical data presented in Fig. 4, these results show that Gpi13 is not essential for M5-DLO scrambling as its deficiency does not impact *N*-glycosylation in a living cell.

## Discussion

We hypothesized that M5-DLO scramblase activity is essential in eukaryotes, and that if it is due to a single protein or unique protein complex, then that protein (or components of the complex) would be essential for cell viability. We devised and implemented an activity correlation profiling approach (Fig. 2) to identify the scramblase and curated the mass spectrometry data (Fig. 3b) to generate a list of 6 M5-DLO scramblase candidates (Fig. 3c), five of which are essential for growth of yeast cells (the sixth candidate, non-essential Gpi7, belongs to the group containing the essential protein candidates Gpi13 and Mcd4 (Fig. 3g)). All six proteins are ER residents with multiple transmembrane spans and well-established functions in GPI anchoring and protein *N*-glycosylation. The latter point raises the interesting possibility that the specific scrambling of M5-DLO across the ER membrane is a moonlighting activity^39,40^ of a known protein.

We tested the role of the six candidate proteins in M5-DLO scrambling using a combination of biochemical and genetic approaches. The biochemical approach is designed to reveal whether a specific protein in a crude mixture of proteins is the sole contributor of activity. Our data indicate that none of our candidates contributes significantly to M5-DLO scramblase activity. Thus, quantitative removal (using genomic TAP-tagging and immunodepletion) of any one of the candidates from a crude mixture of ER membrane proteins (TE) prior to membrane reconstitution did not decrease the potency of the TE to populate an ensemble of large unilamellar vesicles with M5-DLO scramblase (Fig. 4).

We also carried out genetic tests to complement the biochemical assays. We focused on Gpi13, Gpi7 and Mcd4, a family of three GPI P-EtN transferases that catalyze essentially identical reactions (Fig. 3f) and have a similar membrane topology comprising multiple (> 10) transmembrane spans (Fig. 3g). Of considerable interest, all three were shown to participate in a function distinct from their role in GPI anchoring, i.e. ATP secretion^41^. In addition, Mcd4 in particular was shown to have a non-canonical role in aminophospholipid metabolism and ER protein quality control^42,43^. These intriguing moonlighting functions^39,40^ of the GPI P-EtN transferases suggested that each of these three proteins could independently contribute to M5-DLO scramblase activity. In order to identify the potential contributions of each of these equally expressed proteins (Fig. S5) biochemically, we considered extending the results presented in Fig. 4 by performing the scramblase activity assay on proteoliposomes reconstituted over a range of protein-phospholipid ratios and using a Poisson model to detect the change in the size of the M5-DLO pool after biochemical elimination of any one of the three proteins. As noted above, this is a finicky measurement that we deemed would be unlikely to generate new information. We also considered purifying each of the proteins and testing for scramblase activity after reconstitution. Rather than carry out this sufficiency test which brings with it a unique set of issues, e.g. the protein may not be amenable to purification in functional form, we opted instead for a genetic approach. Knowing that *T. brucei* expresses only the Gpi13 ortholog of this 3-member family, we chose to knockout TbGPI13 and ask if we could recover viable cells. Our prediction was that if Gpi13 played an essential role in *N*-glycosylation, i.e. it is the sole M5-DLO scramblase in *T. brucei*, then we would not be able to generate GPI13Δ trypanosomes (as noted above, the GPI anchoring function of Gpi13 is not essential in *T. brucei* cells grown in culture)^45–48^. However, we were able to generate viable GPI13Δ cells that had the expected deficiency in GPI anchoring (Fig. 5c,d), but no evident disruption of protein *N*-glycosylation (Fig. 5e). Thus, Gpi13 does not play an essential role in *N*-glycosylation (similar gene knockouts were also done for TbGPI2 and TbGPI14, resulting in viable cells (AJ, AC, PB, unpublished results) indicating that these proteins too do not play an essential role in *N*-glycosylation).

Despite our inability to identify the M5-DLO scramblase by activity correlation profiling, our results nevertheless reveal unique features of the M5-DLO scramblase that will be critical in guiding future efforts. Furthermore, our results highlight the possibility of using activity profiling as a general method to target the other scramblases of the dolichol pathway required for translocating MPD and GPD. In the following, we consider technical aspects of the profiling approach that would account for our results, highlighting implications for understanding the characteristics of the M5-DLO scramblase.

(1) We detected the vast majority (255) out of 286 known yeast ER membrane proteins (list provided by M. Schuldiner (Weizmann Institute), based on data available at http://www.weizmann.ac.il/molgen/loqate/) (the 31 proteins that we did not detect are listed in Table S3), but the M5-DLO scramblase may have eluded detection. This could be because it might have only a few lysine and/or arginine residues, the possible consequence of having primarily transmembrane sequences with minimal extramembrane loops, and that the resulting long tryptic peptides are not optimal for detection in the mass spectrometer. The relative resistance of M5-DLO scramblase activity to trypsin treatment^16^ is consistent with this possibility.

(2) We filtered the mass spectrometric data by imposing the criterion that M5-DLO scramblase candidates must have ≥ 3 TM spans. Known phospholipid scramblases of the GPCR and TMEM16 protein families have at least 7 TM spans^35,55,56^, consistent with their function as transporters. Indeed, if the M5-DLO scramblase would have fewer than 3 TM spans we would predict that it must homo- or hetero-oligomerize in order to form a functional transport entity.

(3) We hypothesized that M5-DLO scramblase activity is essential and extrapolated this to imply that the activity is due to a single protein and that this protein would therefore also be essential. We considered the possibility that several proteins might individually possess M5-DLO scramblase activity. In this case, the individual proteins may not be essential. Accordingly, we compiled a list of non-essential proteins whose profiles correlate well with the activity profile (Table S4). This list includes 18 ER-localized non-essential proteins with ≥ 3 predicted TM spans, plus 11 other proteins (also with ≥ 3 TM spans) whose subcellular location is not known. It would be interesting in the future to explore the possibility that these proteins may contribute to M5-DLO scramblase activity.

(4) The most intriguing and likely possibility that accounts for our results is that the M5-DLO scramblase co-exists in two or more forms, a complication that could account for the difficulty (noted in the Introduction) in purifying it. A simple example that we term 'X + XY' serves to illustrate this point. Thus, the scramblase (Protein X) co-exists as a monomer that has scramblase activity, and a heterodimer (complexed with Protein Y) that is inactive. The protein profile of Protein X would therefore track the rising portion of the activity profile (corresponding to monomeric Protein X) and then continue more broadly (corresponding to a complex of Protein X and Protein Y) before returning to baseline. The resulting broad profile of Protein X revealed by mass spectrometry would correlate poorly with the activity profile, resulting in Protein X being discarded as a scramblase candidate through our data curation procedure. Examples of potential 'X + XY' profiles (Fig. S7) reveal interesting proteins such as the GlcNAc phosphotransferase Alg7 (R = 0.65 ± 0) (mean ± SD) and the mannosyltransferase Alg2 (R = 0.66 ± 0.05) (mean ± SD), both of which recognize dolichyl diphosphate and could thereby possibly play a role in scrambling M5-DLO. The profiles of both these proteins track the rising portion of the activity profile (fractions 5–7) but then broaden out even as scramblase activity decreases. Both proteins have been reported to form complexes. Thus, Alg2 forms a complex with the mannosyltransferases Alg1 and Alg11^57^, and Alg7 has been reported to form a hexameric complex with Alg13 and Alg14^58^. Although neither of these specific complexes was seen in our data sets, possibly because of differences in sample preparation, the breadth of their protein profiles indicates that Alg2 and Alg7 must nevertheless be involved in some type of complex formation. Thus, Alg2 and Alg7 potentially provide examples of Protein X that exists as a monomer and also in complex with Protein Y(s). More work needs to be done in the future to identify and evaluate such candidates. We note that the 'X + XY' model is the simplest amongst a variety of related scenarios. For example, scramblase activity may be due to a complex of two proteins that are individually inactive—in this situation the protein profiles would diverge from the rising portion of the activity profile, and only track with the activity as it decreases in higher fractions.

In conclusion, our results are best explained by postulating that M5-DLO scramblase activity is due to a protein or proteins whose activity is regulated by complex formation, i.e. at the level of quaternary structure. The simple 'X + XY' model described above serves to illustrate this point, although we note that more elaborate scenarios are possible. The regulation of activity at the level of quaternary structure complicates the use of activity profiling, accounting for our inability to identify the M5-DLO scramblase in this instance. Nevertheless, the approach is broadly applicable and could be used to identify or learn about the MPD and GPD scramblases of the dolichol pathway. For example, the MPD scramblase is amenable to biochemical reconstitution^10,59^ and its activity was successfully enriched by ion exchange fractionation on DEAE-Sephacel^59^. Orthogonal separations by ion exchange and velocity gradient sedimentation could thus be used to generate complementary activity profiles leading to the molecular identification of MPD scramblase using the strategies described here. These approaches form the basis of future work.

## Materials and methods

### Materials

Analytical-grade reagents were purchased from Sigma-Aldrich unless stated otherwise. Antibiotics were from Sigma-Aldrich, Invivogen or Invitrogen. Other reagents/materials were obtained as follows: D-2-[^3^H]-mannose (20–30 Ci/mmol) (American Radiolabeled Chemicals Inc.), Micro BCA protein assay kit (Thermo Fisher Scientific), Triton X-100 (Roche), IgG-Agarose resin (GE Healthcare), anti-TAP antibody (Genescript), anti-Dpm1 antibody (Abcam), SM-2 Biobeads and P6-Biogel resin (Biorad) and egg phosphatidylcholine (Avanti Polar Lipids). Yeast strains used in this study are listed in Table S5.

### Buffers

Buffer A: 10 mM HEPES–NaOH pH 7.4, 100 mM NaCl, 1% (w/v) Triton X-100; Buffer B: 10 mM HEPES–NaOH pH 7.4, 100 mM NaCl; Buffer C: Buffer B supplemented with 3 mM MgCl_2_, 1 mM CaCl_2_, 1 mM MnCl_2_.

### Preparation of [^3^H]M5-DLO

*Protocol 1.* Procyclic form *T. brucei* lacking TbRft1 (TbRft1^−/−^) were generated from *T. brucei* strain Lister 427 and maintained in culture as previously reported^23^. 10^9^ cells were washed with glucose-free SDM-80, resuspended at a density of 1 × 10^8^ cells/ml in the same medium, and incubated for 90 min at 27 °C. D-[2-^3^H]-mannose (200 μCi) was added and the cells were incubated for a further 2 h at 27 °C. The cells were then collected by centrifugation and lipids were sequentially extracted from the washed cell pellet with 3 × 5 ml chloroform/methanol (3:2, v/v), 3 × 5 ml chloroform/methanol/water (3:48:47, v/v/v), containing 1 M MgCl_2_, and 1 × 5 ml chloroform/methanol/water (3:48:47, v/v/v) without MgCl_2_. Finally, [^3^H]M5-DLO was extracted with 2 × 5 ml chloroform/methanol/water (10:10:3, v/v/v). Extracts were pooled, dried under a stream of nitrogen, and partitioned between water and n-butanol. [^3^H]M5-DLO was recovered in the n-butanol phase. *Protocol 2.* Yeast cells (*alg3Δ*) were metabolically labeled with D-[2-^3^H]mannose in low-glucose medium as previously described^16^, and subjected to differential solvent extraction as for Protocol 1 to purify [^3^H]M5-DLO. *Radiochemical purity of [*^*3*^*H]M5-DLO preparations.* The radiochemical purity of [^3^H]M5-DLO preparations was evaluated by thin layer chromatography (TLC) of the intact lipid, and by HPLC analysis of the oligosaccharide portion (Fig. S1). TLC was done on glass-backed Silica Gel 60 plates (Merck) with chloroform/methanol/water (10/10/3, v/v/v) as the solvent system. The chromatograms were visualized using a Raytest Rita* radioactivity TLC analyser (Berthold Technologies). HPLC analysis was carried out by Bobby Ng and Hudson Freeze (Sanford-Burnham-Prebys Medical Discovery Institute, La Jolla, CA) as described^60^. Briefly, the [^3^H]M5-DLO preparation was dried and treated with a 1:1 (v/v) mixture of isopropanol and 0.2 N HCL for 30 min at 100 °C. The released oligosaccharide was analyzed on a Dionex Ultimate 2000 HPLC system using an Agilent Microsorb NH2-column. Radioactivity was detected using an in-line Lablogic Radiodetector and the analysis was calibrated using a co-injected 2-aminobenzamide-labeled dextran ladder (ProZyme/Agilent) detected on a Dionex fluorescence detector.

### Triton extract (TE) enriched in yeast ER membrane proteins

TE was prepared from yeast cells as described previously^61^, except that an additional step was included to salt-wash the membranes prior to detergent solubilization. Briefly, 600 OD_600_ units of cells (BY4741, or BY4741-derived strain expressing a TAP tagged version of the protein of interest) were harvested, washed and homogenized using glass beads. After low-speed centrifugation to clear unbroken cells, the homogenates were centrifuged at 200,000×*g*_*av*_ for 30 min to pellet membranes. The membranes were then resuspended in buffer containing 0.5 M sodium acetate, followed by incubation on ice for 30 min. After re-pelleting, the salt-washed membranes were resuspended in ice-cold Buffer B before being solubilized by gradually adding an equal volume of 2% (w/v) ice-cold Triton X-100 (final concentration 1% (w/v)). The sample was incubated on ice for 30 min before removing insoluble material by centrifugation at 200,000×*g*_*av*_ for 1 h to generate a clear supernatant (TE). We previously used SDS-PAGE immunoblotting to show that TE is enriched in ER membrane proteins such as Dpm1 and Sec61, and depleted of plasma membrane proteins (Pma1, Gas1), as well as proteins of the vacuole (Vph1) and Golgi/endosomes (Pep12)^16^.

### Fractionation of TE by velocity sedimentation on a glycerol gradient

1.5 mL TE was loaded onto a 10 mL continuous glycerol gradient (5% to 25% (w/v), prepared in Buffer A) and centrifuged for 30 h in an SW41 Ti rotor at 100,000×*g*_*av*_. A mixture of sedimentation standards (anhydrase, ovalbumin, bovine serum albumin, β-amylase) was loaded onto a parallel gradient. Fractions were collected manually or using an Auto Densi-Flow apparatus (Labconco). The refractive index of each fraction was measured before removing glycerol by buffer exchange on a desalting spin column packed with Biogel-P6 resin equilibrated in Buffer A. Migration of the sedimentation markers was determined by SDS-PAGE/Coomassie-staining of the fractions.

### Immunodepletion of a candidate protein from TE

TE from yeast strains expressing a TAP-tagged protein of interest (Table S5) was divided into two aliquots. One aliquot was treated at 4 °C for 2 h with IgG-agarose beads to remove specifically the TAP-tagged protein, whereas the other was mock-incubated. Removal of the protein from the extract was verified by SDS-PAGE immunoblotting, using an anti-TAP antibody (1:1,000 dilution) and anti-Dpm1 (1:1,000 dilution). The Dpm1 immunoblot served as a specificity control for immunodepletion, as well as a loading control.

### Quantitative mass spectrometry

Six samples (fractions 5 to 10 of the gradient) were analyzed by mass spectrometry as follows. Proteins in each sample were digested by trypsin after reduction and alkylation with dithiothreitol and iodoacetamide. The resulting peptides were labeled with 6-plex TMT reagents. The digests were combined and subjected to offline fractionation by high pH reversed phase chromatography to obtain 6 fractions. Each fraction was analyzed by LC–MS. Online chromatography was performed with a Thermo Easy nLC 1000 ultra-high-pressure HPLC system (Thermo Fisher Scientific) coupled online to an Orbitrap Fusion Lumos mass spectrometer with a NanoFlex source (Thermo Fisher Scientific). Analytical columns (~ 25 cm long and 75 µm inner diameter) were packed in-house with ReproSil-Pur C18 AQ 3 µM reversed phase resin (Dr. Maisch GmbH, Ammerbuch-Entringen, Germany). A peptide mixture was loaded onto the analytical column with buffer A (0.1% formic acid) and separated with a linear gradient of 3% to 32% buffer B (100% ACN and 0.1% formic acid) at a flow rate of 300 nL/min over 240 min. MS data were acquired using a data-dependent method, dynamically choosing the most abundant not-yet-sequenced precursor ions from the survey scans. Peptide fragmentation was performed via higher energy collisional dissociation. The MS data were processed by the MaxQuant software for protein identification and quantitation. The false discovery rate for protein identification was 1%. A Pearson correlation score (R) was obtained for each identified protein by comparing the measured profile profile and the activity profile.

### Candidate selection

Proteins with R > 0.9 were selected. Using Yeastmine^62^ this list was intersected with the list of all membrane proteins^63^, as well as all essential proteins in *Saccharomyces cerevisiae* (list generated using Yeastmine). This final list was manually completed with the number of transmembrane domains and cellular localization for each protein using information available in the Saccharomyces Genome Database.

### Liposomes and proteoliposome preparation

Vesicles were reconstituted essentially as described^16,20^. Briefly, for each reconstitution sample, [^3^H]M5-DLO (20,000 cpm) was mixed with 4.5 µmol egg PC, dried under a stream of N_2_ and resuspended in 540 µl Buffer B. The lipids were sonicated before being supplemented with Triton X-100 (from a 10% stock solution in water, final concentration 1% (w/v)). For proteoliposomes, TE or a fraction from the velocity gradient was added; for control (protein-free) liposomes, an equivalent amount of Buffer A was added instead. The final volume of the sample was 1 ml. Vesicle formation was induced by removing detergent with SM2 Biobeads (previously washed with methanol and water) added in two stages: 100 mg of the beads were added and the sample was incubated with end-over-end mixing at room temperature; following this incubation, a further 200 mg of the beads were added and vesicles were incubated with end-over-end mixing overnight at 4 °C. The resulting vesicles were pelleted by centrifugation (200,000×*g*_*av*_, 1 h, 4 °C) and resuspended in 200 µl Buffer C. Vesicle protein was estimated using the Kaplan Pederson method^64^ and phospholipid was measured as described previously^65^.

### M5-DLO scramblase assay

The assay was performed as previously described^16,17,20^. Briefly, 20 µl aliquots from a reconstituted liposome or proteoliposome sample were transferred to each of three microfuge tubes, supplemented with either 40 µl of Buffer C (“buffer”), Buffer C containing 3.6 mg/ml Concanavalin A (“ConA”) or Buffer E containing 3.6 mg/ml Concanavalin A and Triton X-100 1.5% (w/v) (“ConA-TX”), and incubated for 30 min on ice. The incubation was terminated by adding 140 µl of blocking solution (bovine serum albumin 20 mg/ml, yeast mannan 2 mg/ml), followed immediately by 1.4 ml of chloroform/methanol (1:1, v/v). The sample was vortexed and centrifuged (20,000×*g*_*av*_, 5 min, 4 °C). The supernatant was transferred to a 20-ml scintillation vial for drying in a fume hood. The pellet was air-dried, then solubilized in 1% (w/v) SDS overnight under vigorous shaking. Radioactivity in the pellet (P) and the supernatant (S) was measured by liquid scintillation counting. For each sample (“buffer”, “ConA” and “ConA-TX”), the percentage of M5-DLO recovered in the pellet fraction was calculated as R = 100•(P)/(P + S). The percentage of M5-DLO flipping was then calculated as 100•(R_ConA_—R_buffer_)/ (R_ConA-TX_—R_buffer_).

### Generation of a TbGPI13 knock out *T. brucei* procyclic strain

Gene disruptions were accomplished in *T. brucei* Cas9-expressing SmOx P9 procyclic forms by replacing both alleles of the target gene with antibiotic resistance genes using the CRISPR/Cas9 technique as described previously^66^. Geneticin and hygromycin replacement cassettes, flanked by 30 bp sequences homologous to the target gene UTRs, were PCR-amplified from pPOTv7 plasmids using gene-specific forward and reverse primers (Table S6). Short guide RNA (sgRNA) templates, consisting of a T7 RNA polymerase promoter sequence and a gene-specific sgRNA sequence to guide double-strand breaks to the UTRs of the target gene, were amplified by annealing gene-specific primers with the G00 sgRNA scaffold primer. All PCR products were purified using Promega Wizard SV PCR clean-up system before transfection. Gene-specific geneticin and hygromycin targeting fragments and 5′ and 3′ sgRNA templates were combined with 1 × TbBSF buffer (90 mM sodium phosphate, 5 mM potassium chloride, 0.15 mM calcium chloride, 50 mM HEPES, pH 7.3) in a total volume of 100 µl and transfected into 3 × 10^7^ SmOx p9 *T. brucei* cells by electroporation using the Amaxa Nucleofector 4D (Lonza), program FI-115. Clonal populations resistant to both hygromycin and geneticin were obtained by limiting dilution, and complete loss of the target gene was verified by diagnostic PCR reactions.

### SDS-PAGE and immunoblotting

*T. brucei* cells were harvested by centrifugation, washed twice with PBS and the pellet suspended directly in SDS sample buffer and heated at 55 °C for 10 min. Approximately 5 × 10^6^ cell equivalents per sample were separated by SDS-PAGE under reducing conditions on a 12% polyacrylamide gel and transferred to nitrocellulose membrane. For all blots, blocking was carried out in 5% milk in TBS, and primary and secondary antibodies diluted in 1% milk in TBS + 0.1% Tween-20 (TBST), with the addition of 0.01% SDS for the secondary antibodies. The following primary antibodies were used: anti-p67 (J. Bangs, 1:5000), anti-ALBA1 (I. Roditi, 1:2500), anti-EP247 (Cedarlane, 1:1500), anti-GPEET K1 (I. Roditi, 1:2500), anti-GPEET 5H3 (I. Roditi, 1:5000) and anti-aldolase (P. Michels, 1:180,000). HRP-conjugated secondary anti-rabbit and anti-mouse antibodies (DAKO) were used at 1:1000 and 1:5000 respectively, and chemiluminescent detection carried out using SuperSignal™ Pico West PLUS substrate (ThermoScientific).

### Flow cytometric analysis

Trypanosomes (~ 5 × 10^6^ cells) were centrifuged and the pellet was directly incubated with one of the following primary antibodies, anti-EP247, GPEET K1 or anti-GPEET 5H3, in SDM-79 media for 30 min. After two washes with SDM-79 samples were incubated with anti-rabbit or anti-mouse AlexaFluor 488 (Invitrogen) secondary antibody (both 1:1000) in SDM-79 for 30 min. Samples were washed two more times and analyzed with a Novo-Cyte flow cytometer (ACEA Biosciences). Samples and buffers were kept on ice throughout, and all centrifugations and antibody incubations were performed at 4 °C.

### Metabolic labeling and extraction of [^3^H]-ethanolamine-labeled GPI precursors and GPI-anchored proteins

Procedures were as previously described^23^. Briefly, 5 × 10^8^ cells were cultured for 16 h in the presence of 40 μCi of [^3^H]ethanolamine. The cells were harvested by centrifugation and washed twice with ice-cold TBS (10 mM Tris–HCl, 144 mM NaCl, pH 7.4). Bulk phospholipids were extracted from the pellet with 2 × 10 ml of chloroform/methanol 2:1 (v/v). GPI precursors and free GPIs were extracted with 3 × 10 ml of chloroform/methanol/water 10:10:3 (v/v/v), and GPI-anchored proteins were extracted with 2 × 10 ml of 9% butanol in water (v/v). GPI precursors and free GPIs were further separated by partitioning the extract between butanol and water. GPI precursors were analyzed by TLC while free GPIs and GPI-anchored proteins were visualized by SDS-PAGE followed by fluorography.

## Supplementary information

Supplementary Table S1.

Supplementary Table S2.

Supplementary Info.
