# Supplementary material for: Complexity of the eukaryotic dolichol-linked oligosaccharide scramblase suggested by activity correlation profiling mass spectrometry
Source: Sci Rep. 2021 Jan 14;11:1411. doi: 10.1038/s41598-020-80956-0 (PMC7809446; doi:10.1038/s41598-020-80956-0)
Supplement: Supplementary file 3 — Supplementary Info. [file 41598_2020_80956_MOESM3_ESM.pdf]

SUPPLEMENTARY INFORMATION

Complexity of the eukaryotic dolichol-linked oligosaccharide scramblase suggested by activity correlation profiling mass spectrometry

Alice Verchère[1], Andrew Cowton[2], Aurelio Jenni[2,3], Monika Rauch[2], Robert Häner[4], Johannes Graumann[5,6], Peter Bütikofer[2*], Anant K. Menon[1*]

[1] Department of Biochemistry, Weill Cornell Medical College, 1300 York Ave, New York, New York 10065, USA
[2] Institute of Biochemistry and Molecular Medicine, University of Bern, Bühlstr. 28, CH-3012 Bern, Switzerland
[3] Graduate School for Cellular and Biochemical Sciences, University of Bern, Mittelstr. 43, CH-3012 Bern, Switzerland
[4] Department of Chemistry and Biochemistry, University of Bern, Freiestr. 3, CH-3012 Bern, Switzerland
[5] Max Planck Institute for Heart and Lung Research, W.G. Kerckhoff Institute, Ludwigstr. 43, D-61231 Bad Nauheim, Germany
[6] German Centre for Cardiovascular Research (DZHK), Rhine-Main site, Max Planck Institute for Heart and Lung Research, Bad Nauheim, Germany

Correspondence:
Anant K. Menon: Department of Biochemistry, Weill Cornell Medical College, 1300 York Ave, New York, New York 10065, USA; E-mail: akm2003@med.cornell.edu
Peter Bütikofer: Institute of Biochemistry and Molecular Medicine, University of Bern, Bühlstr. 28, CH-3012 Bern, Switzerland; E-mail: peter.buetikofer@ibmm.unibe.ch

This document contains supplementary figures and tables as follows:

- Supplementary Figures S1 - S7: pages 2-5
- Supplementary Tables S1 and S2: descriptive information for Table S1 and Table S2 is provided in this document on page 6. The Tables are provided separately as .csv (comma separated value) files.
- Supplementary Tables S3 - S6: pages 7-10

Supplementary Figures

Verchère et al.
Figure S1

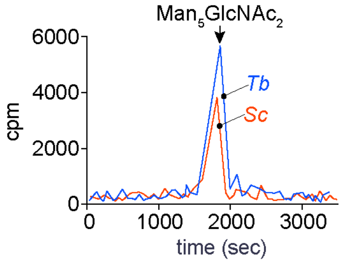

Figure S1. HPLC analysis of the oligosaccharide portion of [^{3}H]M5-DLO. The pyrophosphate linkage in [^{3}H]M5-DLO was hydrolyzed by mild acid treatment, and the released [^{3}H]oligosaccharide was analyzed by HPLC using an amino-column. Representative profiles (radioactivity (cpm) versus elution time) of samples of [^{3}H]M5-DLO prepared via Protocol 1 (*Tb*) and Protocol 2 (*Sc*) are shown. The peak in each case co-elutes approximately with the glucose-9 oligomer in a dextran ladder that was co-injected with the samples. The data were generated by Bobby Ng and Hudson Freeze (Sanford-Burnham-Prebys Medical Discovery Institute, La Jolla, CA, USA).

Verchère et al.
Figure S2

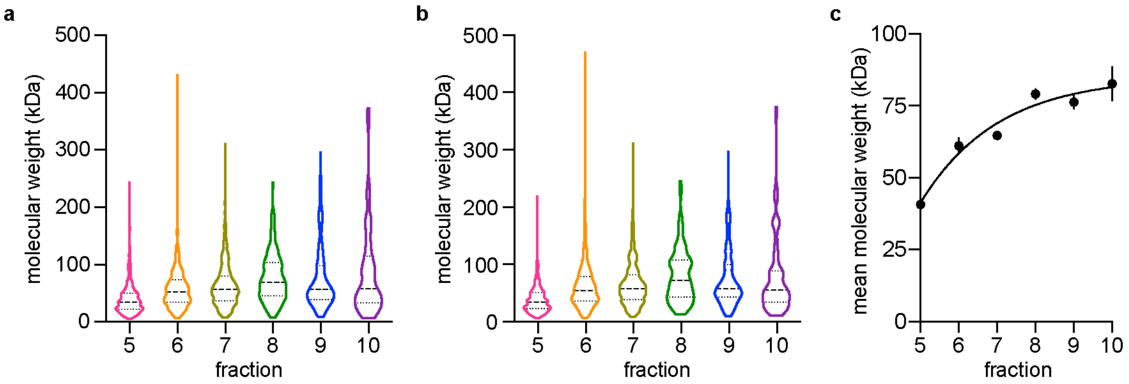

Figure S2. Distribution of proteins in the velocity gradient. The mass spectrometry data were used to locate the peak fraction for each identified protein, i.e. the fraction in which the protein is maximally abundant. The molecular weights of the proteins were obtained from the YeastMine webserver.

a, b. Box and violin plots showing the molecular weights of proteins peaking in each fraction for the two technical replicates of the mass spectrometric analysis. The median and quartiles of the data are indicated.

c. Mean molecular weights of proteins maximally abundant in each fraction. The points show the mean (± standard deviation) of data obtained from two technical replicates. The line through the points is intended to guide the eye.

Verchère et al.
Figure S3

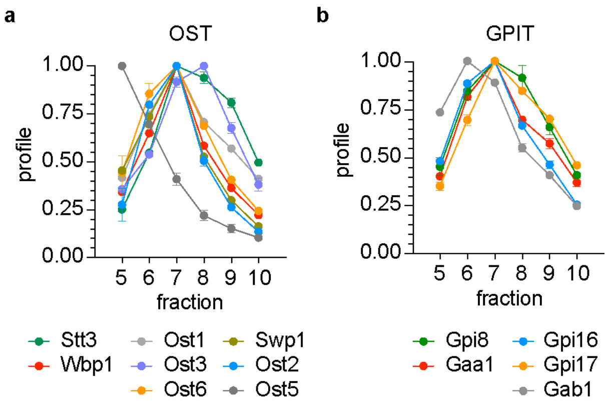

Figure S3. Profiles of OST and GPIT subunits in the velocity gradient. The distribution of subunits of the oligosaccharyltransferase (OST) complex (panel **a**) and the glycosylphosphatidylinositol (GPI) transamidase (GPIT) complex (panel **b**) in the velocity gradient were obtained from the mass spectrometry data. Yeast OST exists in two isoforms that contain either Ost3 or Ost6, in addition to seven other subunits: Stt3, Ost1, Wbp1, Swp1, Ost2, Ost4, Ost5. These complexes can be isolated from yeast membranes after solubilization in dodecylmaltoside detergent supplemented with cholesteryl hemisuccinate (Wild et al. *Science* **359**: 545-550 (2018)). In panel **a**, Triton X-100-solubilized OST complex remains largely intact, with the exception of the Ost5 subunit which sediments less rapidly than the rest of the subunits and appears to have dissociated from the complex.

Verchère et al.
Figure S4

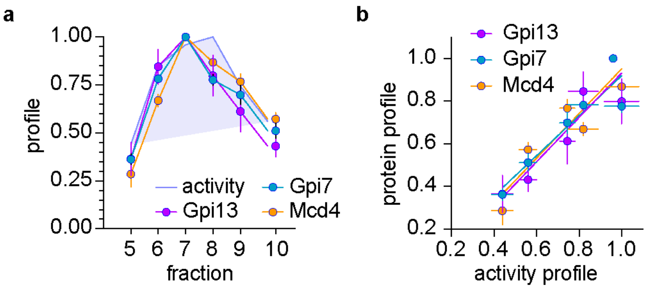

Figure S4: Correlation between the activity profile and the profiles of Gpi13, Gpi7 and Mcd4.
a. Reproduced from Fig. 3d.
b. Correlation between the M5-DLO scramblase activity profile, and profiles of Gpi13, Gpi7 and Mcd4 (taken from panel A). Values defining the profile of a specific protein, i.e. relative protein abundance in a particular fraction, are plotted against the corresponding values of activity and analyzed by simple linear regression. Linear regression yielded R squared values of >0.81, corresponding to R values of >0.90. The plot shows intuitively that activity values are high when relative protein abundance values are high, and correspondingly that activity values are low when relative protein abundance values are low.

Verchère et al.
Figure S5

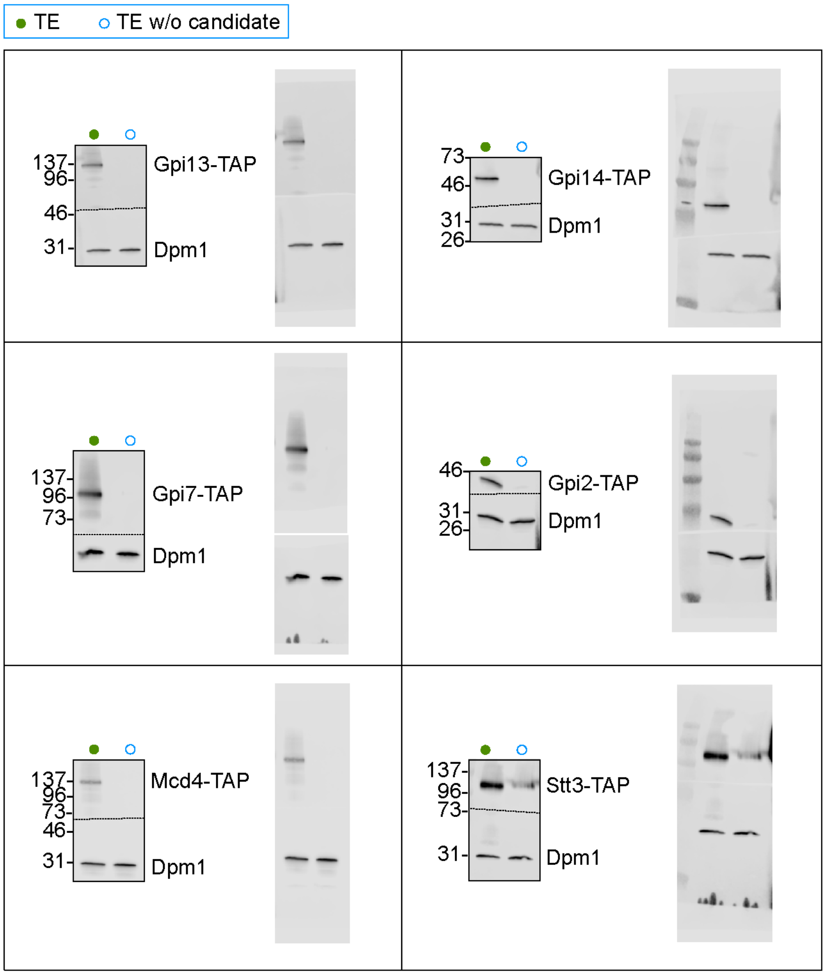

Figure S5: Uncropped immunoblots corresponding to Figure 4. The annotated blots in each panel are reproduced from Figure 4 of the main paper. On the right of each annotated blot is shown the corresponding uncropped original. In all cases the transfer membrane was cut to separate the upper (>40 kDa) and lower (<40 kDa) portions. These were probed separately using antibodies against the TAP tag (upper portion) or the ER membrane protein Dpm1 (lower portion).

Verchère et al.
Figure S6

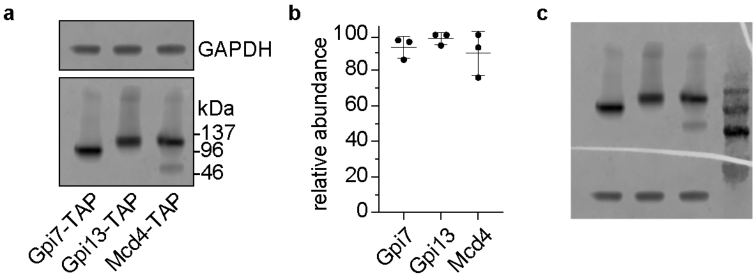

Figure S6: Gpi7, Gpi13 and Mcd4 are expressed at similar levels.

a. 6 OD units of yeast cells expressing chromosomally TAP-tagged Gpi7, Gpi13 or Mcd4 were precipitated with trichloroacetic acid. The pellets were washed with acetone, resuspended in loading dye and analyzed by SDS-PAGE/immunoblotting using anti-TAP and anti-glyceraldehyde phosphate dehydrogenase (GAPDH) antibodies, the latter serving as a loading control.

b. The intensity of bands detected by anti-TAP and anti-GAPDH was quantified using Image Studio software. The ratio of intensities was taken as an indicator of abundance of the TAP-tagged protein. Data for three analyses are shown (mean ± standard deviation).

c. Uncropped blot corresponding to panel **a**.

Verchère et al.
Figure S7

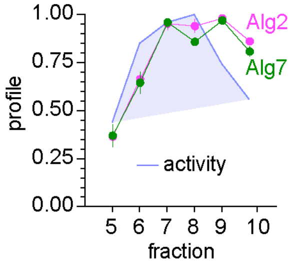

Figure S7: Profiles of Alg2 and Alg7.

Profiles of Alg2 and Alg7 illustrate the possibility that the activity correlation profile method may be complicated by complex formation according to the 'X + XY' model described in the Discussion section.

Table S1
Table S2
Technical replicates of TMT mass spectrometric analysis of velocity gradient fractions (see Fig. 3a and 'Materials and methods' for details). A description of the column headings used in the Tables is given below. The Tables are provided separately as .csv (comma separated value) files.

Column Name	Description
Protein IDs	Identifier(s) of protein(s) contained in the protein group.
Protein names	Name(s) of protein(s) contained within the group.
Gene names	Name(s) of the gene(s) associated to the protein(s) contained within the group.
FASTA headers	FASTA headers(s) of protein(s) contained within the group. FASTA file is a text-based format containing protein sequences, in which amino acids are represented using single-letter codes.
Peptides	The total number of peptide sequences associated with the protein group (i.e. for all the proteins in the group).
Sequence coverage [%]	Percentage of the sequence that is covered by the identified peptides of the best protein sequence contained in the group.
Q-value	This is an indicator of the confidence for protein identification, with range of 0-0.01 (1%). A smaller Q-value represents higher confidence of protein identification.
Columns H-M	Summed up intensity of all the identified peptide sequences
cor_score	The correlation score is the Pearson correlation between the measured profile and the standard profile (see Fig. S4)

Table S3

Predicted ER membrane proteins that were not identified in the present study

Gene	Protein	Function	TM spans
YLL055W	YCT1	Yeast Cysteine Transporter	10
YLR004C	THI73	THI regulon	7
YLR023C	IZH3	Implicated in Zinc Homeostasis	7
YMR274C	RCE1	Ras and a-factor Converting Enzyme	7
YHR140W			6
YER140W	EMP65	ER Membrane Protein of 65 kDa	5
YKR053C	YSR3	Yeast Sphingolipid Resistance	5
YFR042W	KEG1	Kre6-binding ER protein responsible for Glucan synthesis	4
YLR246W	ERF2	Effect on Ras Function	4
YNL008C	ASI3	Amino acid Sensor-Independent	4
YNL194C			4
YLL014W	EMC6	ER Membrane protein Complex	3
YIL089W			2
YFR041C	ERJ5	Endoplasmic Reticulum located J-protein	2
YDR437W	GPI19	Glycosyl PhosphatidylInositol anchor biosynthesis	2
YGR105W	VMA21	Vacuolar Membrane Atpase	2
YLL052C	AQY2	AQuaporin from Yeast	2
YNL046W			2
YNL146W			2
YOR044W	IRC23	Increased Recombination Centers	2
YPL096C-A	ERI1	ER-associated Ras Inhibitor	2
YAL028W	FRT2	Functionally Related to TCP1	1
YBL100C			1
YBR162W-A	YSY6		1
YDL232W	OST4	OligoSaccharylTransferase	1
YLR238W	FAR10	Factor ARrest	1
YNL087W	TCB2	Three Calcium and lipid Binding domains (TriCalBins)	1
YOR324C	FRT1	Functionally Related to TCP1	1
YPL200W	CSM4	Chromosome Segregation in Meiosis	1
YER053C-A			1
YMR122W-A	NCW1	Novel Cell Wall protein	1

Table S4

Non-essential membrane proteins identified with R>0.9 and ≥3 TM spans

ORF	Protein		MW (Da)	TM spans
ER localized				
YDR205W	MSC2	Meiotic Sister-Chromatid recombination	80578.2	13
YBR132C	AGP2	high-Affinity Glutamine Permease	67261.4	12
YJL212C	OPT1	OligoPeptide Transporter	91630.2	12
YOR067C	ALG8	Asparagine-Linked Glycosylation	67409.4	12
YGR227W	DIE2	Derepression of ITR1 Expression	61811	11
YEL031W	SPF1	Sensitivity to Pichia Farinosa killer toxin	135259.6	10
YOR002W	ALG6	Asparagine-Linked Glycosylation	62798.3	10
YFL025C	BST1	Bypass of Sec Thirteen	117747	8
YGL160W	AIM14	Altered Inheritance rate of Mitochondria	65849.6	7
YGL167C	PMR1	Plasma Membrane ATPase Related	104554.8	7
YLR130C	ZRT2	Zinc-Regulated Transporter	46342.2	7
YBR287W			47493.6	6
YJR015W			58116.5	6
YBL011W	SCT1	Suppressor of Choline-Transport mutants	85693.2	4
YKR067W	GPT2	Glycerol-3-Phosphate acylTransferase	83674.9	4
YOR085W	OST3	OligoSaccharylTransferase	39487.6	4
YER072W	VTC1	Vacuolar Transporter Chaperone	14381.3	3
YGR089W	NNF2		106476.7	3
Unknown subcellular location				
YDL206W			85973.1	12
YOL103W	ITR2	myo-Inositol TRansporter	66715.8	12
YLR241W	CSC1	Calcium permeable Stress-gated cation Channel	89348.1	11
YOL158C	ENB1	ENteroBactin	66989.4	11
YER060W	FCY21	FluoroCYtosine resistance	58041.4	10
YHR078W			63331.2	8
YGR199W	PMT6	Protein O-MannosylTransferase	88018.7	8
YGR149W	GPC1	GlyceroPhosphoCholine acyltransferase	51652.9	7
YLR214W	FRE1	Ferric REductase	78879.7	7
YLR443W	ECM7	ExtraCellular Mutant	50314.2	4
YOR301W	RAX1	Revert to Axial	50160.1	3

Table S5
Yeast strains used in this study

Strain	Genotype	Source
GPI13-TAP	*MATa his3Δ1 leu2Δ0 met15Δ0 ura3Δ0 GPI13-TAP::HIS3MX6*	Brenda Andrews
GPI7-TAP	*MATa his3Δ1 leu2Δ0 met15Δ0 ura3Δ0 GPI7-TAP::HISMX6*	Dharmacon[1]
MCD4-TAP	*MATa his3Δ1 leu2Δ0 met15Δ0 ura3Δ0 MCD4-TAP::HISMX6*	Dharmacon[1]
GPI2-TAP	*MATa his3Δ1 leu2Δ0 met15Δ0 ura3Δ0 GPI12-TAP::HISMX6*	Dharmacon[1]
STT3-TAP	*MATa his3Δ1 leu2Δ0 met15Δ0 ura3Δ0 STT3-TAP::HISMX6*	Dharmacon[1]
GPI14-TAP	*MATa his3Δ1 leu2Δ0 met15Δ0 ura3Δ0 GPI14-TAP::HISMX6*	Brenda Andrews
BY4741	*MATa his3Δ1 leu2Δ0 met15Δ0 ura3Δ0*	Brenda Andrews
YG248	*MATa ade2-101 ura3-52 his3Δ200 lys2-801 Δalg3::HIS3*	Markus Aebi

[1]Dharmacon TAP-Fusion ORF collection (strains were confirmed by colony PCR)

Table S6

Primers used to create a TbGPI13 knock out *T. brucei* procyclic strain

Primer	Sequence
TbGPI13-pPOT-Fwd	AGAAGTGTGAGCACTAGACGCTGACTACCCGTATAATGCAGACCTGCTGC
TbGPI13-pPOT-Rev	ACCATCAGCATCAACAACAACACACAAAGACCGGAACCACTACCAGAACC
TbGPI13-sgRNA-5’	GAAATTAATACGACTCACTATAGGCTTTCAGCAAAGTTACACACGTTTTAGAGCTAGAAATAGC
TbGPI13-sgRNA-3’	GAAATTAATACGACTCACTATAGGAAAAAAGGATGACGAGAACAGTTTTAGAGCTAGAAATAGC
G00	AAAAGCACCGACTCGGTGCCACTTTTTCAAGTTGATAACGGACTAGCCTTATTTTAACTTGCTA<br>TTTCTAGCTCTAAAAC

Gene-specific sequences are underlined
